# Long-read sequencing identifies a common transposition haplotype predisposing for *CLCNKB* deletions

**DOI:** 10.1186/s13073-023-01215-1

**Published:** 2023-08-23

**Authors:** Nikolai Tschernoster, Florian Erger, Stefan Kohl, Björn Reusch, Andrea Wenzel, Stephen Walsh, Holger Thiele, Christian Becker, Marek Franitza, Malte P. Bartram, Martin Kömhoff, Lena Schumacher, Christian Kukat, Tatiana Borodina, Claudia Quedenau, Peter Nürnberg, Markus M Rinschen, Jan H. Driller, Bjørn P. Pedersen, Karl P. Schlingmann, Bruno Hüttel, Detlef Bockenhauer, Bodo Beck, Janine Altmüller

**Affiliations:** 1 Cologne Center for Genomics (CCG), University of Cologne, Faculty of Medicine and University Hospital Cologne, Cologne, Germany; 2 Institute of Human Genetics, Faculty of Medicine and University Hospital Cologne, University of Cologne, Kerpener Str. 34, 50931 Cologne, Germany; 3 Center for Molecular Medicine Cologne (CMMC), University of Cologne, Faculty of Medicine and University Hospital Cologne, Cologne, Germany; 4 Department of Pediatrics, Cologne Children’s Hospital, Cologne, Germany; 5 Department of Renal Medicine, UCL, University College London, London, UK; 6 Department II of Internal Medicine, University of Cologne, Cologne, Germany; 7 Department of Pediatrics, University Marburg, Marburg, Germany; 8 FACS & Imaging Core Facility, Max Planck Institute for Biology of Ageing, Cologne, Germany; 9 Max Delbrück Center for Molecular Medicine in the Helmholtz Association (MDC), Hannoversche Straße 28, 10115 Berlin, Germany; 10 Department of Biomedicine, Aarhus University, Aarhus, Denmark; 11 Aarhus Institute of Advanced Studies, Aarhus University, Aarhus, Denmark; 12 Department III of Medicine, University Medical Center Hamburg-Eppendorf, Hamburg, Germany; 13 Department of Molecular Biology and Genetics, Aarhus University, Universitetsbyen 81, DK-8000 Aarhus C, Denmark; 14 Department of General Pediatrics, University Children’s Hospital, Münster, Germany; 15 Max Planck Genome-Centre Cologne, Max Planck Institute for Plant Breeding Research, Cologne, Germany; 16 Great Ormond Street Hospital for Children, NHS Foundation Trust, London, UK; 17 Berlin Institute of Health at Charité - Universitätsmedizin Berlin, Core Facility Genomics, Berlin, Germany

**Keywords:** Bartter syndrome type 3, Salt-wasting tubulopathy, Long-read sequencing, Target enrichment, *CLCNKA*, *CLCNKB*, Structural variant, Risk haplotype, Next-generation sequencing, HiFi-sequencing

## Abstract

**Background:**

Long-read sequencing is increasingly used to uncover structural variants in the human genome, both functionally neutral and deleterious. Structural variants occur more frequently in regions with a high homology or repetitive segments, and one rearrangement may predispose to additional events. Bartter syndrome type 3 (BS 3) is a monogenic tubulopathy caused by deleterious variants in the chloride channel gene *CLCNKB*, a high proportion of these being large gene deletions. Multiplex ligation-dependent probe amplification, the current diagnostic gold standard for this type of mutation, will indicate a simple homozygous gene deletion in biallelic deletion carriers. However, since the phenotypic spectrum of BS 3 is broad even among biallelic deletion carriers, we undertook a more detailed analysis of precise breakpoint regions and genomic structure.

**Methods:**

Structural variants in 32 BS 3 patients from 29 families and one BS4b patient with *CLCNKB* deletions were investigated using long-read and synthetic long-read sequencing, as well as targeted long-read sequencing approaches.

**Results:**

We report a ~3 kb duplication of 3′-UTR *CLCNKB* material transposed to the corresponding locus of the neighbouring *CLCNKA* gene, also found on ~50 % of alleles in healthy control individuals. This previously unknown common haplotype is significantly enriched in our cohort of patients with *CLCNKB* deletions (45 of 51 alleles with haplotype information, 2.2 kb and 3.0 kb transposition taken together, *p*=9.16×10^−9^). Breakpoint coordinates for the *CLCNKB* deletion were identifiable in 28 patients, with three being compound heterozygous. In total, eight different alleles were found, one of them a complex rearrangement with three breakpoint regions. Two patients had different *CLCNKA/CLCNKB* hybrid genes encoding a predicted *CLCNKA/CLCNKB* hybrid protein with likely residual function.

**Conclusions:**

The presence of multiple different deletion alleles in our cohort suggests that large *CLCNKB* gene deletions originated from many independently recurring genomic events clustered in a few hot spots. The uncovered associated sequence transposition haplotype apparently predisposes to these additional events.

The spectrum of *CLCNKB* deletion alleles is broader than expected and likely still incomplete, but represents an obvious candidate for future genotype/phenotype association studies.

We suggest a sensitive and cost-efficient approach, consisting of indirect sequence capture and long-read sequencing, to analyse disease-relevant structural variant hotspots in general.

**Supplementary Information:**

The online version contains supplementary material available at 10.1186/s13073-023-01215-1.

## Background

Bartter Syndrome (BS), first reported in 1962 [1], is unarguably the prototypic Mendelian salt-losing tubulopathy, characterized by defective salt reabsorption in the thick ascending limb (TAL) of Henle and/or the distal convoluted tubule (DCT), resulting in chronic hypokalaemia, hypochloraemia, metabolic alkalosis, and hyperreninaemic hyperaldosteronism with low or normal blood pressure [2]. BS forms a clinically heterogenous spectrum with variable onset and severity manifesting from antenatal life to adulthood with variable clinical signs (with/without nephrocalcinosis), and sometimes extrarenal findings like sensorineural deafness [1, 3, 4]. Today, the BS spectrum is genetically classified into five subtypes (types 1–5) and Gitelman syndrome (GS; phenotypically defined by hypomagnesemia and hypocalciuria in addition to hypokalaemic metabolic alkalosis) [1, 4–15]; however, there is wide phenotypic overlap between the BS and the GS (like) spectrum [2]. BS 1–3, and 4a, as well as *SLC12A3*-associated GS constitute autosomal recessive disorders. The ultra-rare BS 4b follows digenic recessive inheritance, and *MAGED2*-associated BS 5 is an X-linked recessive disorder (Additional file 1: Table S1).

BS 3 is caused by biallelic pathogenic variants in the *CLCNKB* gene encoding the ClC-Kb chloride channel [8]. There are more than 140 different causative sequence variants reported in the *CLCNKB* gene (HGMD database [16] accessed in 12/2022), but complete deletions of *CLCNKB* account for more than 50% of all BS 3 disease alleles, often found in a homozygous state [8, 17–21]. The *CLCNKB* gene is directly adjacent to the highly homologous *CLCNKA* gene (94% coding sequence identity [8]), presumably as a result of an ancient gene duplication. This genomic structure likely predisposes the locus to meiotic rearrangements, as is known to happen to other similar structured loci (e.g. the *CYP11B1/CYP11B2* locus [22]).

Clinically, the BS 3 phenotype seems the most variable of all BS types, ranging from antenatal/neonatal BS (30 %), classic infantile/childhood BS (44 %), to a GS-like phenotype (26 %) [17, 20, 23–25] in the 115 patients analysed in a recent retrospective French study [20]. Patients with BS 3 are at risk to develop chronic kidney disease (CKD), which can progress to kidney failure in some cases. Preliminary data on genotype/phenotype correlations for BS 3 indicate that deletions and truncating mutations are associated with earlier diagnosis and higher risk for CKD [20, 23]. Currently, genetic diagnosis is commonly performed with Multiplex Ligation-dependent Probe Amplification (MLPA), a method that does not allow to determine the size of the SV and detection of breakpoint regions of the deletion.

In this study, using a combination of synthetic long-read sequencing and Nanopore/PacBio Long-read Third Generation Sequencing (LRS), we performed an in-depth structural investigation of the *CLCNKA*/*CLCNKB* locus. Analysing 32 patients with *CLCNKB* deletion-associated BS 3, and one patient with digenic BS 4b (P17), we shed new light on the genomic complexity of this rearrangement-prone region.

## Methods

### Patients

We identified 33 patients from 30 families (32 individuals with BS 3, and 1 with BS4b) with a diagnosis of Bartter syndrome and a deletion of *CLCNKB* on at least one allele, who had genetic testing performed at the laboratories at the departments of nephrology and paediatric nephrology at the University Hospital of Cologne (Family 1 and P18), Great Ormond Street Hospital in London (P2-P14), and the department of paediatrics at the University of Marburg, together with the department of general paediatrics at the University Children's Hospital of Münster (P15-P17, and P19-P30). Clinical data were obtained from the treating clinicians using a standardized questionnaire. Appropriate informed consent was obtained using protocols approved by the respective local research ethics committees.

Long-range PCR was attempted for all 33 samples and the product was sequenced using LRS in 24 patients. Whole genome LRS was performed in 3 patients, targeted enrichment LRS in 4 patients, and linked-read WGS in 1 patient (some samples were analysed multiple times with different technologies) [26]. For a summary of the molecular genetic analyses performed on each patient, see Additional file 1: Table S2. Patient P18 was previously diagnosed with a presumably hemizygous 5 bp deletion in exon 9 of *CLCNKB* resulting in a frameshift (c.847_851delTTCTT; p.Phe284Cysfs*38, Additional file 2: Fig. S1). Patient P17 has previously been reported by Schlingmann et al. with digenic BS 4b [11].

### High molecular weight DNA isolation

High molecular weight (HMW) genomic DNA isolation for linked read and Xdrop applications was performed using the MagAttract HMW DNA Kit (Cat. No. 67563) for 200 μl fresh EDTA blood input according to the manufacturer’s specifications (Qiagen, Hilden, Germany).

### Variant reporting

Genomic coordinates, unless otherwise stated, refer to the hg19 human genome reference sequence. Descriptions of cDNA or protein changes refer to the RefSeq and UniProt references NM_004070.3 and P51800 (*CLCNKA*), and NM_000085.4 and P51801 (*CLCNKB*). As a consequence of the high sequence homology between *CLCNKA* and *CLCNKB*, the breakpoints in a rearrangement between both loci may—if the breakpoints lie in a nucleotide stretch with complete sequence identity—be impossible to localize to a single nucleotide. In these cases, breakpoint regions are specified.

### Whole-exome sequencing (WES)

WES was performed using Agilent SureSelect Whole Exome v7 enrichment (Agilent Technologies, Santa Clara, CA, USA), followed by NGS on an Illumina NovaSeq 6000 platform (Illumina, San Diego, CA, USA). Data analysis and NGS-based CNV detection were performed using the Cologne Center for Genomics Varbank2 application v.3.3 [27] (Cologne Center for Genomics, Cologne, Germany). In particular, we filtered for high-quality (coverage >15-fold; Phred-scaled quality >25), rare (minor allele frequency (MAF) ≤0.01) variants. To exclude pipeline-related artefacts, we additionally filtered against common variants from in-house WES datasets.

### 10× Genomics linked-read analysis

High molecular weight DNA was extracted for library preparation following the Chromium Genome Reagent Kit standard protocol (CG00022 RevA) using the Chromium Genome Chip Kit PN-120216 (10× Genomics, Pleasanton, USA) and the Genome Library, Gel Bead & Multi- plex V1 Kit PN-120229 (10× Genomics) with the modification of using 0.9 ng of genomic DNA input. The fragment size of the prepared library was assessed using Tapestation 4200 (Agilent Technologies). The library was sequenced on a NovaSeq6000 sequencer (Illumina), which generated 1.63×10^9^ paired-end reads. Assembly was performed using the de novo genome assemblies setting of the Long Ranger v.2.2.2 genome assembler (https://github.com/10XGenomics/longranger) and visualized using the Loupe Browser v.2.1.1 [28]. 96.4% of reads were mapped, reaching a mean coverage depth of 70.3×.

### Samplix Xdrop indirect sequence capture and ONT long-read sequencing

The Xdrop indirect sequence capture allows the isolation of specific genomic DNA fragments which contain a region of interest (ROI) and enriches these fragments for long-read sequencing applications. Essentially, a small known genomic sequence of ~ 150 bp (detection sequence) is used near the ROI to select genomic high molecular weight DNA fragments of up to 50 kb spanning the breakpoint region or other structural variants of interest. To identify the breakpoint regions of the *CLCNKB* deletion, Xdrop indirect sequence capture was performed in selected patients (Additional file 1: Table S2). High molecular DNA is encapsulated with a PCR reaction mix (Samplix, Birkerød, Denmark). The short fluorescence-labelled detection sequence (forward primer; 5′-ATCCTGACACAGCCATCTGC-3′ and reverse primer; 5′-TGATCACGCAGAACCCTCAG-3′) is used to mark our ROI (Additional file 2: Fig. S2). Droplets containing the genomic ROI were enriched using a FACS Aria IIIu (BD Biosciences, Franklin Lakes, USA) using the 100-micron nozzle at 20 psi pressure, gating based on forward scatter pulse height, side scatter pulse height, and droplet fluorescence pulse height. Droplets were sorted using the “Yield” precision mode for the best possible recovery of droplets of interest. The sorting of droplets is described in more detail by Madsen et al. [29]. Before sequencing, the evaluation sequence (forward primer; 5′-GCCCAGAAGAGTTATGTGGCT-3′ and reverse primer; 5′-GAGCCCTTGGAAAGCGAGTA-3′) (Additional file 2: Fig. S2) is used to assess the enrichment factor as a quality control. DNA is released from the isolated droplets and encapsulated again containing a multiple displacement amplification mix for target enrichment followed by long-read sequencing using a GridIon sequencing device from Oxford Nanopore (Oxford Nanopore Technologies, Oxford, UK). Sequencing reads were aligned using the minimap2 software v2.17 [30], with the pre-specified map-ont parameter. The resulting alignment files were sorted and indexed using samtools v1.7 [31] and visualized in the Integrative Genomics Viewer software v.2.10.2 [32].

### Long-range polymerase chain reaction (PCR) and amplicon-based SMRT sequencing

Specific forward primer 5′-AGATACTGGTTTTCCGTCATCTC-3′ and reverse primer 5′-TACCTTTGTGGATATTTCCTCCTAC-3′ were designed to exclusively amplify a ~6450 bp region covering the *CLCNKB* breakpoint regions previously identified by Xdrop targeted enrichment as described above. PCR was performed with 100 ng gDNA and 0.4 μM of primers using the LA-Taq polymerase and 2xGC PCR-Buffer I according to manufacturer protocols (Takara Bio Inc., Kusatsu, Japan).

### PacBio whole genome long-read sequencing (single-molecule real-time SMRT-Seq)

Libraries were prepared using the SMRTbell Express Template Prep Kit 2.0 (PN 101-853-100, Pacific Biosciences, Menlo Park, CA, USA). Briefly, 5 μg of the genomic DNA was sheared to 15 kbp (Megaruptor 3, Diagenode, Denville, New York, USA), followed by performing damage repair, end repair, A-tailing and hairpin adapter ligation and final exonuclease treatment. All libraries were size selected using BluePippin (Sage Sciences, Beverly, MA, USA) running with a size cut-off of 10,000 bp. AMPure PB magnetic beads (Pacific Biosciences) were used for all purification steps. Library size and quality were assessed using Fragment Analyzer (Agilent Technologies) and Qubit fluorometer with Quant-iT dsDNA HS Assay Kits (Invitrogen, Waltham, MA, USA).

Sequencing primer v5 and Sequel 2.2 DNA Polymerase were annealed and bound, respectively, to the final SMRTbell library. Libraries were loaded at an on-plate concentration of 80 pM and sequencing was performed using 8M SMRT cells on the Sequel II System with Sequel II Sequencing Plate 2.0 for 24h movie time. Secondary analysis (HiFi reads) was performed using Pacific Biosciences SMRT Link v10.2. The sequencing data were then processed and analysed as described above.

### Analysis of CLCNKA/CLCNKB expression data

Expression data for *CLCNKA* and *CLCNKB* were downloaded from The Cancer Genome Atlas’ Pan-Cancer Atlas [33] for all 510 renal clear cell carcinoma samples. Expression RSEM values were then analysed in R (v4.2.2) using the R packages tidyverse [34] (v2.0.0), car [35] (v3.1-2), and goft [36] (v1.3.6). For all performed analyses, expression outliers were removed and RSEM values ≤ 50 were considered (83.1% and 81.2% of observations for *CLCNKA* and *CLCNKB*, respectively). For the calculation of the gamma distribution goodness-of-fit, zero expression values were also excluded.

Plots were generated using base R’s ggplot() and the car package’s qqPlot() function. Significance values for goodness-of-fit analyses were calculated with the shapiro.test() function for the normal and lognormal distributions, and with the goft package’s gamma_test() and gamma_fit() functions for the gamma distribution.

A *p*-value of ≥0.05 was considered to signify that the gene’s expression distribution was not significantly different from the respective statistical distribution category and thus compatible with the gene’s expression pattern following this distribution.

### ClC-Kb/ClC-Ka hybrid protein structure predictions

Protein structures of reference and hybrid ClC protein sequences were predicted using the ParaFold [37] version of Alphafold2 [38] using default parameters. Since ClC channels are homodimeric, the homodimeric states were generated using the multimer pre-set (*n*=2).

## Results

### Genetic workup of Family P1

**Fig. 1:**
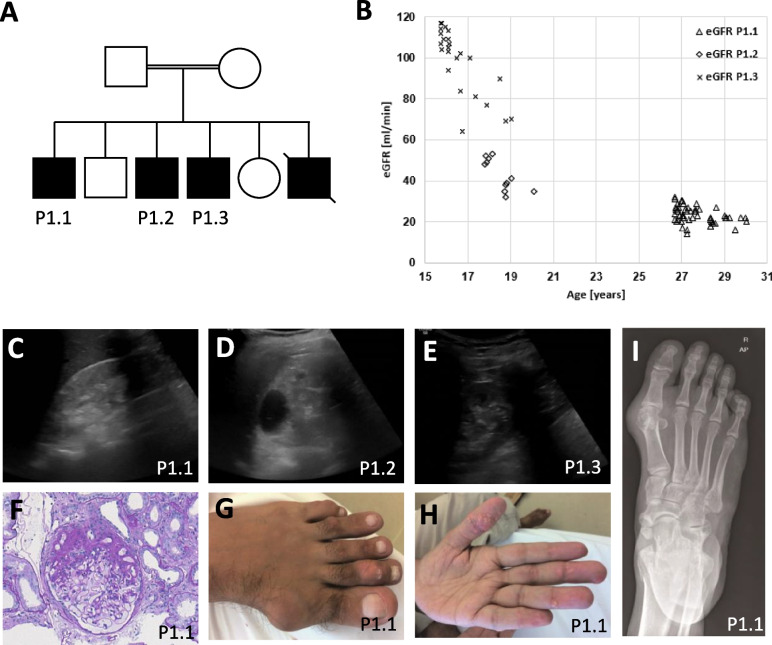
Clinical findings and pedigree in severely affected family P1. **A** Pedigree of family P1. **B** Estimated glomerular filtration rate (eGFR) in all brothers over a 15 years’ timeline. Note that eGFR declines over age in all brothers. **C**–**E** Discordant kidney sonograms. **C** Increased echogenicity, nephrocalcinosis, and reduced cortico-medullary differentiation. **D** Increased echogenicity, nephrocalcinosis, and multiple large cysts. **E** Mildly increased echogenicity. **F** Histologic slide of kidney biopsy showing unspecific focal segmental glomerulosclerosis in P1.1. **G**–**I** Destructive gouty arthritis and multiple gout tophi in P1.1

**Fig. 2:**
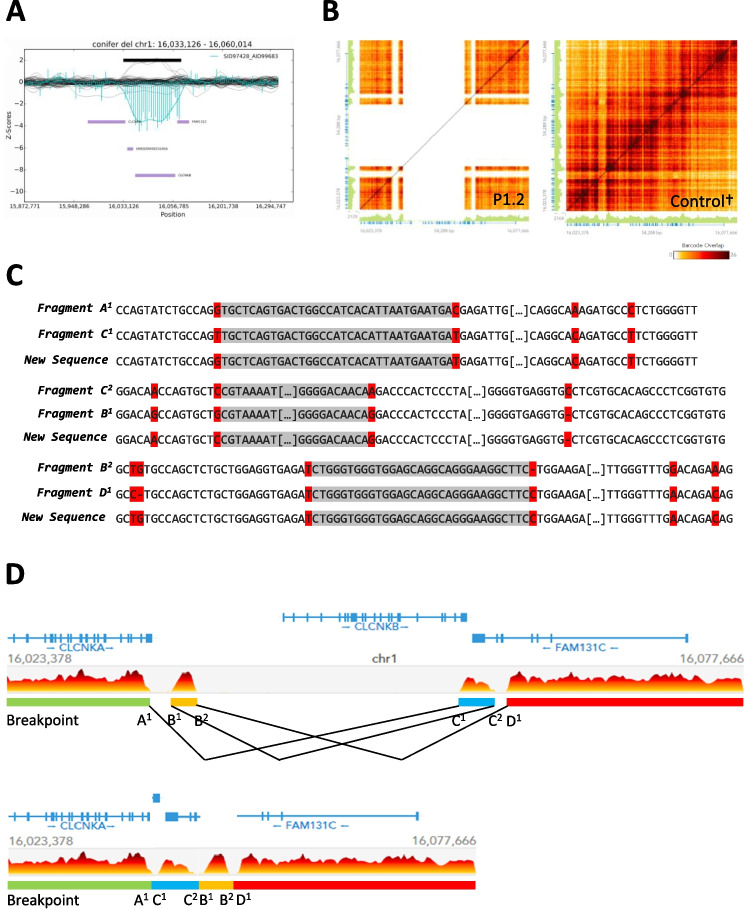
Results of the WGS and linked-read WGS analysis from patient P1.2. **A** Varbank2 implemented conifer CNV analysis tool showing results from the WGS dataset. *Z*-scores from patient P1.2 in cyan-coloured bars and also as extrapolated curve. Affected region is indicated by the black horizontal bar. Genes in this region are indicated by purple horizontal bars. The markers (probes throughout the exons) are evenly distributed among the positional *X*-axis. The black curves represent the control collective, consisting of a fixed set of samples for the given enrichment kit. **B** Linked-read Loupe browser v.2.1.1 haplotype resolved SV results for patient P1.2 compared to a control sample (matrix view). Genomic region chr1:16.023.378-16.077.660 (hg38) is displayed on *y*- and *x*-axis for both alleles, respectively. Number of reads is indicated by green vertical bars. Sequence coverage (barcode overlap) is indicated by the heat map ranging from zero coverage (white) to high coverage (black). **C** Xdrop indirect sequence capture and ONT long-read sequencing results for patient P1.2 [26]. Gene-specific nucleotides are indicated by red background; breakpoint regions are indicated by grey background. Long stretches of homologous genomic DNA sequence have been shortened, indicated by […]. **D** Reconstruction of the genomic structural rearrangement of patient P1.2. The three breakpoint regions result in a rearrangement of fragment B and fragment C and the deletion of the genomic DNA in between these fragments. Genomic DNA Fragments were rearranged according to the identified breakpoints. New genomic rearrangement consisting of fragment A-C-B-D. The common sequence transposition haplotype equates to the transposition of fragment C. Genes are indicated in blue with exons as blue vertical bars. Gene orientation is indicated by blue arrows. Colour coding of genomic fragments A (green), B (orange), C (blue), and D (red). Genomic sequence coordinates refer to the hg38 human reference. † Linked-read control carries the *CLCNKA* 3′ UTR sequence transposition haplotype heterozygously

Family P1 first presented in the paediatric nephrology department in 2018 with three male siblings with hypokalaemic metabolic alkalosis, hypochloraemia, hyperuricaemia, and progressive chronic kidney disease (Fig. 1A–F). WES of the three patients revealed no causative single nucleotide variants, but WES-based CNV detection suggested a homozygous deletion of *CLCNKB* (Fig. 2A). A region of homozygosity (ROH) value of 389 Mb confirmed the reported consanguinity. Due to the unusually severe clinical presentation of the oldest sibling with intradermal tophi and gouty arthritis (Fig. 1G–I), we performed breakpoint detection using linked-read whole-genome sequencing [26]. We identified an unusual pattern consisting of a large homozygous deletion encompassing *CLCNKB* and two smaller adjacent deletions nearby, but the precise genomic architecture remained unclear due to discontinuous sequencing reads (Fig. 2B). As our attempts to identify breakpoints by PCR were unsuccessful, we used Xdrop indirect sequence capture and subsequent ONT long-read sequencing in patient P1.2 [26]. Here, we identified three breakpoint regions between 30 and 410 bp in size that could not be further narrowed down because of their sequence homology (Fig. 2C). The genomic architecture was then reconstructed as shown in Fig. 2D. Based on the peculiar genomic findings in Family P1, we initiated a more detailed characterisation of the complete locus in a cohort of *CLCNKB* deletion patients.

### Results of the genomic workup of the cohort

Long-range PCR amplifying a ~6450 bp DNA fragment covering the genomic breakpoint regions found in patient P1.2 was performed, and 29 additional patients with *CLCNKB* deletion-associated BS 3 and one patient with BS 4b were analysed [26]. The long-range PCR yielded an amplicon in 25 of the 33 patients. In total, we generated long-read sequence data of 27 patients. For six patients, no sequence data were obtained (P1.1 was not sequenced; see Additional file 1: Table S2). Clinical data for all patients included in this study are summarized in Table 1.

**Table 1:** Patient cohort

**Family patient ID**	**Sex**	**Age at study**	**Age at definite diagnosis**	**Country of origin**	**Phenotype**	**Other complications, remarks**	**eGFR (ml/min/1.73m**^**2**^**) or serum creatinine (mg/dl) on last follow up**	**Deletion alleles (letter code)**
P1.1	Male	31	0	Syria; c	ABS	Destructive gouty arthritis	<15 (age 31 y)	C
P1.2	Male	22	0	Syria; c	ABS	Hyperuricemia	35 (age 22 y)	C
P1.3	Male	20	0	Syria; c	ABS	Hyperuricemia, seizures (normal EEG)	70 (age 19 y)	C
P2	Female	46	nd	UK-Asian	CBS	Short statue	27	E
P3	Female	18	1	UK-Asian	CBS	(polyhydramnios, but born 42 wks Presented with critical collapse with multisystem organ failure age 1 y in the context of enteroviral infection	70 (age 17 y)	E
P4	Male	15	7	UK-Asian	CBS	Childhood, initially presented age 2 y with growth failure, referred to nephrology age 7 y with persistent hypokalaemia	140 (age 8 y)	A
P5	Male	15	8	UK-black	GS	Childhood, presented age 7 y with abdominal pain and blood tests showed hypokalaemia/hypomagnesaemia	155 (age 10 y)	F
P6	Female	10	3	UK-Asian	CBS	Childhood, presented age 3 y with growth failure and noted to have hypokalaemia and alkalosis	180 (age 10 y)	H
P7	Female	32	~6	Italy	CBS, GS	Presented initially in Italy, notes only say that she presented initially as CBS, but then looked more and more like GS, so that BS3 was suspected.	102 (age 17 y)	B
P8	Male	18	2	Sri Lanka	ABS	Antenatal, (polyhydramnios, born at term), 3 siblings died in infancy of unclear cause in Sri Lanka	60 (age 17 y)	B
P9	Male	29	1	UK-black	ABS	Antenatal, (27 wk gestation), nephrocalcinosis, nephrotic range proteinuria (first noted age 13), quantified as 3.3 g/d	67 (age 16 y)	B, F
P10^a^	Female	10	1	UK-white	nd	Born 34 wk (no polyhydramnios) presented age 4 days with weight loss, found to have HSD3B2 deficiency.	120 (age 1 y)	D
P11	Male	nd	1	Italy	ABS	Antenatal (polyhydramnios with 4 amnioreductions, born at term), intrauterine growth restriction, birthweight 2130g. In neonatal period noted to have hypoklaemic alkalosis	95 (age 3 y)	G
P12	Female	17	1	UK-Iranian	ABS	Antenatal (polyhydramnios, but born at term), presented at age 3 months with growth failure and severe electrolyte abnormalities: Na 120, K: 1.0)	50 (age 17 y)	E
P13	Male	29	7	Iran	CBS	Childhood, pre-school age presentation with growth failure, polyuria, hypokalaemia history of previous fetal loss with polyhydramnios	15 (creatinine 350, age 18 y)	E
P14	Female	3	1	UK-black	CBS	Presentation age 2 months with growth failure, noticed to have hypokalaemia	110 (age 3 y)	B, F
P15.1^b^	Male	nd	nd	German, f	nd	5 wk	nd	A
P15.2^b^	Male	nd	nd	German, f	nd	Childhood, age 4 years	nd	A
P16^b^	Male	nd	nd	Turk, f	nd	5 wk	nd	nd
P17^b, c^	Female	34	nd	Turk, c	ABS	Antenatal, (polyhydramnios, born at 28 wks gestation), polyuria, hypokalaemia, metabolic alkalosis, sensorineural deafness, digenic BS 4b	nd	B
P18	Female	29	22 mo	Congo, s	CBS	Childhood, pre-school age presentation with growth failure	90 (age 27 y)	F
P19	Female	27	2.5 mo	Turk, c	CBS	Infantile onset	Creatinine 1.3 (age 4.5 y)	nd
P20	Male	nd	nd	Turk, c	nd	nd	nd	B
P21	Male	23	3 wk	Turk, c	CBS	Antenatal (34 wk gestation) vomiting, hypokalaemic alkalosis	Creatinine 0.9 (age 14 y)	E
P22	Male	21	5 mo	Tamil	CBS	Infantile onset, vomiting, hypokalaemic alkalosis	Creatinine 0.3-0.4 (age 1 y)	nd
P23	Female	24	9 mo	France, s	CBS	Infantile onset, failure to thrive, polyuria	nd	F
P24	Female	20	5 wk	Arabia, c	CBS	Infantile onset	Creatinine 0.4 (age 2 mo)	E
P25	Female	32	nd	Rwanda, s	nd	nd	nd	nd
P26	Female	19	6 mo	Afghanistan, c	CBS	nd	nd	nd
P27	Male	18	2 mo	Turk, c	CBS	Failure to thrive	nd	E
P28	Female	16	nd	Turk, c	ABS	nd	nd	E
P29	Female	nd	nd	Turk, c	nd	nd	nd	E
P30	Male	nd	nd	Africa, s	CBS	nd	nd	B

*ABS*, antenatal BS; *CBS*, classical BS; *GLS*, Gitelmann-like syndrome; lower case letters in the country of origin column indicate c, consanguineous; familial (f); sporadic (s) case; *wk*, week; *mo*, months; *y*, years; *nd*, no data

^a^Patient has been previously reported in Giri et al. [39]

^b^Patients have been previously reported in Konrad et al. [17]

^c^Patient has been previously reported in Schlingmann et al. [11]

**Fig. 3:**
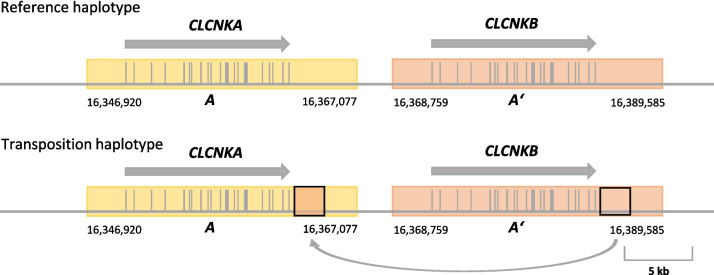
Genomic *CLCNKA/CLCNKB* locus and the common *CLCNKA* 3′ UTR sequence transposition haplotype on chromosome 1p36.13. The genomic environment surrounding the *CLCNKA/CLCNKB* gene locus contains two highly homologous genomic regions (A and A’, sequence homology approx. 80 %). A and A’ are indicated by the yellow and orange boxes, respectively. The homologous region A stretches over 20.157 bp from chr1:16,346,920-16,367,077 (GRCh37/Hg19), region A’ stretches over 20.827 bp from chr1:16,368,759-16,389,585 (GRCh37/Hg19). The exonic sequence homology of *CLCNKA* and *CLCNKB* is 94% [8]. In the alternative transposition haplotype, a 2.2–3-kb large DNA fragment of the *CLCNKA* 3′ UTR (small orange box, arrow head) is replaced by the homologous fragment from the 3′ UTR of *CLCNKB* (small orange box, arrow tail), resulting in the loss of the genomic material downstream of *CLCNKA* and an extra copy of the DNA fragment duplicated from the *CLCNKB* 3′ UTR. Gene length and orientation of *CLCNKA* and *CLCNKB* are indicated by the grey arrows. Exons are indicated as grey bars

Patient P11 with no long-range PCR product was first analysed using Xdrop targeted DNA enrichment and long-read sequencing, identifying the breakpoint regions. In this patient, the first breakpoint region is located upstream of the long-range PCR forward primer resulting in the loss of the PCR forward primer binding site. Of the other seven patients with no long-read PCR amplicons, two patients (P6 and P10) were subsequently analysed using whole genome long-read sequencing, which identified the cause of amplification failure [26]. In patient P10, one breakpoint region is located 1727 bp downstream of the long-range PCR reverse primer binding site, thus deleting the binding site. In patient P6, a single breakpoint region located 302 bp upstream of the long-range PCR forward primer binding site resulted in the loss of the binding site. Taken together, we identified the breakpoint regions in 28 of the 33 patients with *CLCNKB* deletions. Whereas we initially hypothesized that the deletions in the *CLCNKA*/*CLCNKB* locus largely represent complex events, our investigation of the larger cohort and healthy controls revealed the presence of a common, but previously unreported structural haplotype. In most patients, we detected a transposition of a 2.2–3.0-kb long segment of the human genome reference *CLCNKB* 3′ untranslated region (UTR) to the corresponding region in the *CLCNKA* 3′ UTR. The longer 3.0-kb transposition haplotype covers a sequence in which the *CLCNKA* and *CLCNKB* references differ in only 105 nucleotides (sequence identity approx. 96 %). The smaller 2.2-kb transposition haplotype covers a sequence in which the *CLCNKA* and *CLCNKB* references differ in only 75 nucleotides (sequence identity also approx. 96 %). Both haplotypes share the same 5′ breakpoint region but have significantly different 3′ breakpoint regions clearly distinguishing both haplotypes. In NGS datasets aligned against the reference genome, the presence of these transpositions results in an apparent loss of *CLCNKA* 3′ UTR sequence with a concomitant gain of *CLCNKB* 3′ UTR genetic material (see Fig. 3). Forty-five (88.2 %) of the structurally characterized deletion alleles for which a haplotype determination could be made (alleles A-F, *n*=51) lie on the variant haplotype. Additionally, we reviewed the *CLCNKA*/*CLCNKB* gene cluster in the new T2T CHM13-v2.0 human reference genome [40] of our patients and confirmed both the sequence transposition haplotype as well as the different deletion alleles (Additional file 2: Fig. S3).

In our review of a small cohort of non-BS short-read and long-read whole genome in-house control datasets, we found this sequence transposition haplotype to be common (10/22 alleles and 5/18 alleles, respectively), indicative of a high population frequency. Based on coverage data from more than 76,000 whole-genome sequencing analyses in the gnomAD v3.1.2 dataset, we estimate the worldwide allele frequency of the variant haplotype at approximately 50% (2.2-kb and 3.0-kb transposition taken together; Additional file 2: Fig. S4). An analysis of *CLCNKA* and *CLCNKB* expression levels in a large dataset of renal clear cell carcinoma samples from The Cancer Genome Atlas’ (TCGA) Pan-Cancer Atlas database [33] showed no evidence of a pronounced effect of the common transposition haplotype on gene expression. Both genes’ expression patterns are compatible with a gamma distribution (*CLCNKA p*-value 0.0504, *CLCNKB p*-value 0.4076), the second most common type of expression pattern distribution in TCGA datasets after the normal distribution [41]. A bimodal or multimodal expression pattern, as might be expected if the common haplotype had a marked effect on gene expression, was not seen (Additional file 2: Fig. S5).

To investigate whether the human reference haplotype or the transposition haplotype is the ancestral haplotype, we compared the human reference sequence to the rhesus monkeys’ and other, more distantly related species’ reference sequences. While the rhesus monkey reference sequence corresponds to the human reference sequence in 40 of the allele-defining 69 nucleotides (58 %), a meaningful comparison was not feasible in other studied species, likely due to larger genetic distance. The moderate reference sequence similarity between human and rhesus monkey does not currently prove or rule out the presence of either haplotype in the rhesus monkey population, and we thus cannot determine the ancestral haplotype.

**Fig. 4:**
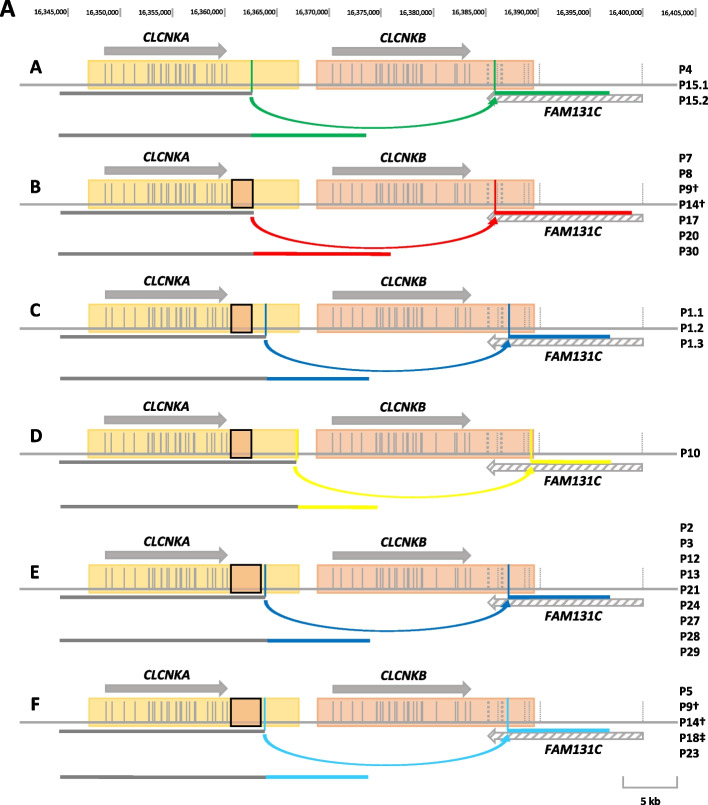
Summary of the *CLCNKB* deletion alleles identified in this study. Breakpoint regions are indicated by coloured vertical lines. Corresponding breakpoint regions are indicated by coloured arrows. Identical breakpoint regions are coloured in the same colour. Two common *CLCNKA* 3′ UTR sequence transpositions identified in this study are indicated by the orange box within the yellow box of *CLCNKA*. Breakpoint region coordinates of the 2.2 kb and 3 kb sized sequence transpositions are listed in Additional file 1: Table S3. Exons are indicated as vertical bars. Orientation and length of *CLCNKA* and *CLCNKB* are indicated by grey arrows. The gene *FAM131C*, located on the negative strand, is indicated by the grey-hatched arrow and is only partially visualized. *FAM131C* exons are indicated by dotted lines. **A** Deletion allele A is derived from the reference haplotype. Deletion alleles B, C, and D are derived from the smaller 2.2 kb sized sequence transposition haplotype. Deletion alleles E and F are derived from the larger 3 kb sized sequence transposition haplotype. Adjacent breakpoint region coordinates that discriminate the deletion alleles E and F are listed in Additional file 1: Table S3. All patients show the corresponding deletion allele in a homozygous state if not indicated otherwise. **B** Deletion alleles G and H that result in *CLCNKA*/*CLCNKB* hybrid genes. Since the haplotype-defining genomic sequence is deleted on these alleles, a haplotype determination could not be made. † Patients, compound heterozygous for the deletion alleles B and F; ‡ Heterozygous deletion allele. This patient carries a 5 bp deletion in exon 9 *in trans*

Altogether, we found eight different deletion alleles (termed here A-H; Fig. 4). At least five of the eight deletion alleles (B, C, D, E, F) derive from the variant haplotype (Fig. 4). Allele A derives from the reference haplotype. The origin of deletion alleles G and H cannot be determined with certainty based on sequence data, as the deletion spans the haplotype-defining sequence segment. Interestingly, we could detect alleles of various complexity ranging from simple single breakpoint regions to more complex “scattered” alleles with three breakpoint regions (allele G). Deletion alleles C and E are defined by the same breakpoint region, but differ in the length of the variant haplotype sequence transposition segment in the *CLCNKA* 3′ UTR (2.2 kb vs. 3.0 kb). A summary of each patient’s breakpoint region coordinates can be found in Additional file 1: Table S3. The breakpoint region in allele C and E is the most common breakpoint region identified in this study (24 of 56 alleles), with allele E being the single most common allele (18 of 56). All SVs found in this study are in a homozygous state, except for patients P9, P14, and P18. P9 and P14 both carry the deletion alleles B and F compound heterozygously (Fig. 4A). Sequence overviews of all deletion alleles found in our cohort are shown in Additional file 2: Fig. S6.

### CLCNKA/CLCNKB hybrid gene

In two patients, we found breakpoint regions affecting the coding sequence of both *CLCNKA* and *CLCNKB*. Patient P11 carries the complex deletion allele G with three breakpoint regions. The first breakpoint region is located in a homologous region of 53 bp in intron 7 of *CLCNKA* and intron 7 of *CLCNKB* resulting in an in-frame fusion of *CLCNKA* exons 1–7 to *CLCNKB* exons 8–20. The second and third breakpoint regions are located in the 3′ UTR of *CLCNKA* and are not predicted to have an effect at the protein level. Patient P6 carries a single breakpoint in a homologous region of 144 bp in intron 15 of *CLCNKA* and intron 15 of *CLCNKB* resulting in an in-frame hybrid gene composed of exons 1–15 of *CLCNKA* and exons 16–20 of *CLCNKB* (Fig. 4B).

### Predicted ClC-Ka/ClC-Kb hybrid proteins

**Fig. 5:**
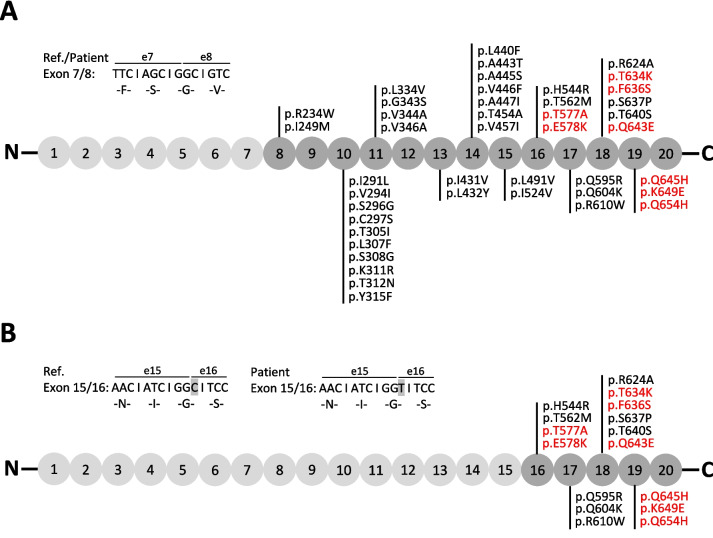
Workup of the ClC-Ka/ClC-Kb hybrid genes in patients P6 and P11. ClC-Ka AA-sequence is indicated in light grey; ClC-Kb AA-sequence is indicated in dark grey; AA changes between wildtype ClC-Ka and ClC-Ka/ClC-Kb hybrid protein are annotated in the corresponding exon. AA changes analysed in detail in Figure 6 are shown in red. **A** Hybrid gene in patient P11. Breakpoint region in intron 7/8. Genomic sequence at the border between exon 7 and exon 8 divided into triplets and the corresponding AA are shown. Ser218 is the last AA encoded by exon 7 before the breakpoint region. **B** Hybrid gene in patient P6. Breakpoint region in intron 15/16. Genomic sequence at the border between exon 15 and exon 16 divided into triplets and the corresponding AA are shown. I540 is the last AA encoded by exon 15 before the breakpoint region. AA G541 is encoded by the last two nucleotides of exon 15 and the first nucleotide of exon 16. In the hybrid gene, the first nucleotide in exon 16 encoded by *CLCNKB* has changed from C>T but the corresponding AA G541 remains unchanged in the hybrid protein

**Fig. 6:**
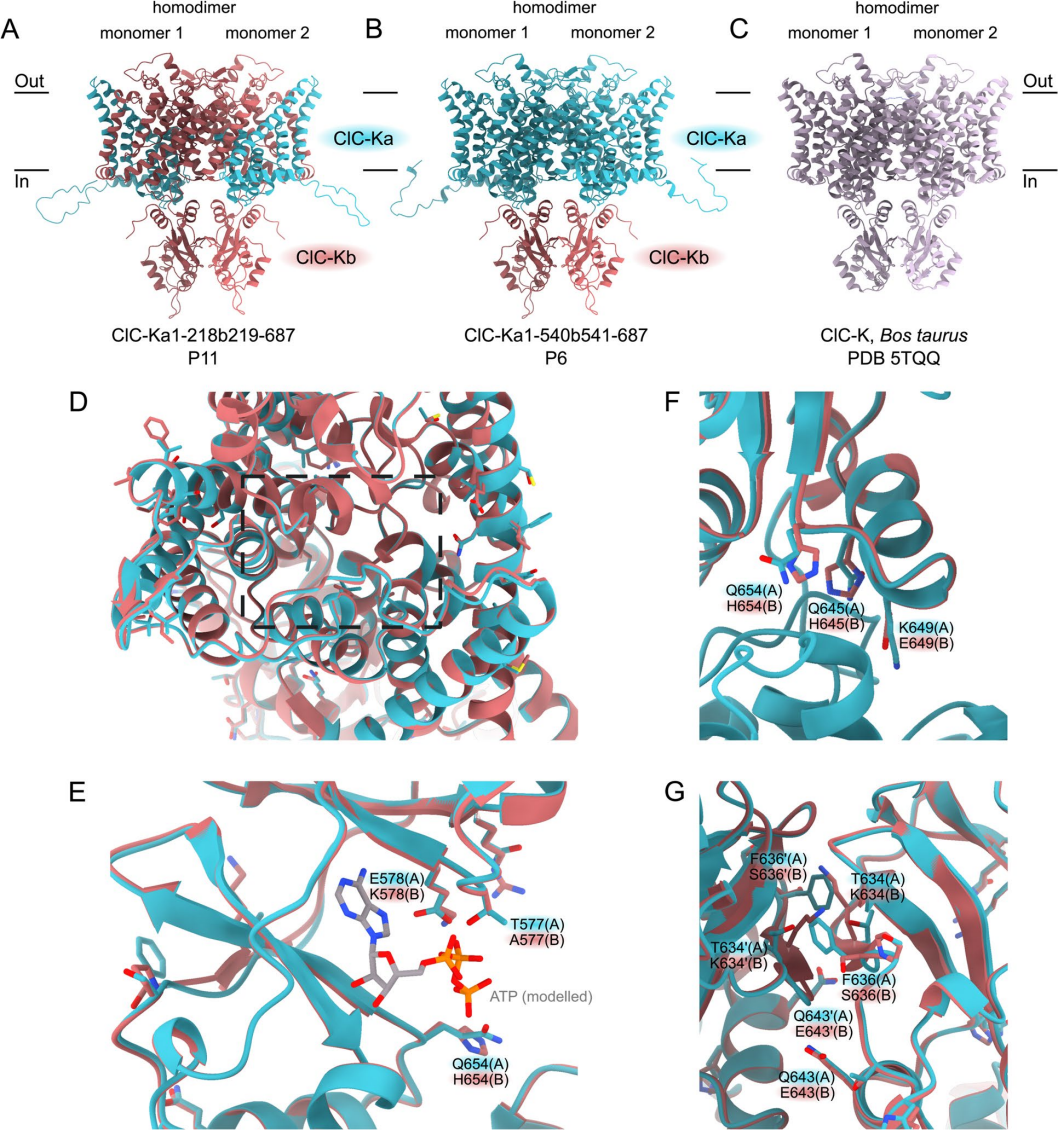
Predicted ClC-Ka/ClC-Kb hybrid proteins in patients P6 and P11. **A** AlphaFold2 prediction of the ClC-Ka1-218b219-687 hybrid homodimer (P11). **B** AlphaFold2 prediction of the ClC-Ka1-540b541-687 hybrid homodimer (P6). **C** Cryo-EM structure of the ClC-K homodimer from Bos taurus (PDB ID: 5TQQ, 84,3% seq ID to ClC-Ka and ClC-Kb). **D** View on the membrane region of ClC-Ka (teal) and ClC-Kb (pastel red) with the amino acid variations shown in sticks shows no significant changes in the ion tunnel between the variants ClC-Ka and ClC-Kb. **E** Putative adenosine nucleotide binding site in ClC-Ka and ClC-Kb superimposed with an ATP bound in ClC-7 structure (Protein data base: 7jm7). **F** Interface between the cytosolic CBS domains of the ClC-Ka and ClC-Kb proteins. **G** Dimerization interface of the CBS domains in the ClC-Ka and ClC-Kb shows distinct differences

ClC-Ka and ClC-Kb channels share a high sequence identity of 91.3%. The hybrid genes identified in patients P6 and P11 result in two different ClC-Ka/ClC-Kb hybrid proteins. In patient P11, the predicted hybrid protein is composed of amino acids (AA) 1–218 of ClC-Ka and AA 219–687 of ClC-Kb (Figs. 5A and 6A). Due to the high sequence identity between ClC-Ka and ClC-Kb, this hybrid protein differs in 43 AA positions from the wildtype ClC-Ka protein. In patient P6, the breakpoint is located at Gly541, behind the last transmembrane helix of ClC-Ka. The predicted hybrid protein is composed of AA 1–540 of ClC-Ka and AA 541–687 of ClC-Kb altering the ClC-Ka AA-sequence by 16 AA (Figs. 5B and 6B).

As no frameshifts are introduced or deletions occur in the hybrid genes, the overall topology of ClC-K channel is retained in these hybrids and we assume that the hybrid genes express stable proteins. We predicted the structures of ClC-Ka and ClC-Kb as well as the two hybrids in a homodimeric state using Alphafold2 (AF) [37, 38]. The AF predictions show minimal difference between the two homologs and the two hybrids with a root-mean-square deviation (RMSD) between Cα atoms of the structures of 0.3Å (see Fig. 6A, B). We checked the validity of the predictions by comparing to the cryo-EM structure of ClC-K from Bos Taurus (PDB ID: 5TQQ, 84,3% seq. ID to ClC-Ka and ClC-Kb). The predictions were found to be highly similar to the Bos Taurus ClC-Channel with an RMSD of 0.5 Å, supporting that the predictions are correct [42] (see Fig. 6A–C). As AA variations in the hybrid proteins arise from the two endogenous proteins, most AA variations exhibit the same sidechain properties (e.g. hydrophobic:hydrophobic, polar:polar) within the membrane helices. Since the tunnel of the protein is not affected by any AA variation between ClC-Ka and ClC-Kb, interference with channel function would thus not be expected (Fig. 6D).

Interestingly, both hybrids contain the cytoplasmic cystathionine beta-synthase (CBS) domain of the ClC-Kb channel, which are known regulators in ClC proteins via binding to adenosine nucleotides thereby activating or inhibiting channel function [43]. This could suggest that the activity of the hybrid proteins is regulated as the ClC-Kb would be. The CBS domains of ClC-Ka and ClC-Kb show distinct differences in the putative adenosine nucleotide binding side (577 Thr(A)/Ala(B), 578 Glu(A)/Lys(B), 654 Gln(A)/His (B)) when superimposing the ATP bound in ClC-7 (RCSB Protein data base 7jm7) to the predicted structures (Fig. 6E). Binding of ATP in ClC-7 is however also supported by the N-terminal region of the protein, which is missing from our models as we cannot predict ligand binding [44]. It is currently still unknown if ClC-K proteins are regulated by adenosine nucleotides as other ClC proteins are. Several interesting AA changes can also be found in the CBS domain interface with the membrane region (Fig. 6F) and the CBS dimerization interface (Fig. 6G).

## Discussion

*CLCNKB* deletions are the leading cause of Bartter syndrome type 3 worldwide [8, 18, 19, 21].

In this study, we report the *CLCNKB* deletion breakpoint region coordinates of 27 patients from 24 families with BS 3, and one patient with BS 4b. We characterize the deletion alleles utilizing long-read sequencing in 27 patients (24 long-range PCR LRS, 3 whole genome LRS, 4 targeted enrichment LRS, and 1 linked-read WGS; some samples were analysed multiple times with different technologies) and detected eight different *CLCNKB* deletion alleles, which are likely caused by non-allelic homologous recombination (NAHR) with homologous flanking regions >200 bp as suggested by Ebert et al. [45]. For the remaining five patients with no long-read PCR amplicons, additional molecular genetic workup was not performed. For two of these patients, the DNA quality was very low, which could explain the failure to generate a long-range PCR product. Given the number of different deletion alleles detected in this study, the probability of additional genotypes is high.

Based on these results, we estimate that the long-range PCR reported here is capable of identifying about 75% (49/65) of the deletion alleles, assuming that all patients with a single deletion allele type are homozygous for this deletion allele. This procedure cannot exclude compound heterozygosity for a “long-range PCR positive” deletion allele with a “long-range PCR negative” deletion allele. However, such a constellation was not detected in any patient analysed by whole-genome LRS or indirect sequence capture LRS (*n*=7). The discrimination between two different “long-range PCR positive” deletion alleles in the same patient (as in patients P9 and P14) is possible. A fully sensitive and specific as well as allele-agnostic method is the indirect sequence capture approach by Xdrop followed by LRS, or alternatively whole-genome LRS—the latter being cost-prohibitive in most settings. In our view, a reasonable procedure to precisely detect breakpoint alleles is to start with a screening long-range PCR followed by indirect sequence capture LRS if no PCR product can be derived.

The presence of multiple different deletion alleles in our cohort suggests that *CLCNKB* whole gene deletions originated from many independently recurring genomic events.

This in-depth approach characterizing *CLCNKB* deletion alleles may prove useful for the investigation of genotype/phenotype correlations in BS 3 patients, who show a remarkable phenotypic variability that currently remains largely unexplained. Approximately 1/3 of patients carrying biallelic *CLCNKB* deletions present antenatally with polyhydramnios [8, 17, 24, 46]. This observation is consistent with the clinical data of our cohort, in which 34% (9 of 26) of patients for which phenotype data is available also had an antenatal onset of disease (see Table 1). Whether the precise determination of the deletion allele has any clinical impact on patient care (association with onset of disease and/or disease progression (e.g. kidney failure)) cannot yet be predicted given our cohort size and study design (cross-sectional genomic study with no longitudinal phenotype data in most patients). This question needs to be revisited in larger BS 3 cohorts with corresponding longitudinal clinical data. The *CLCNKB* deletions also include parts of the 3′ UTR of *FAM131C*. The size of the *FAM131C* 3′ UTR deletion was not associated with the differences concerning the clinical phenotype in our cohort. Furthermore, no monogenic disease caused by mutations in *FAM131C* is known to date and *FAM131C* has not been attributed to any particular function.

*CLCNKB* full gene deletions are most often described as homozygous [17–19, 21, 39, 46]. Only a few studies reported cases with heterozygous whole *CLCNKB* gene deletions in combination with other mutations [19, 47, 48]. Cases of two different whole gene *CLCNKB* deletion alleles have not been reported until now. This is most likely because previous studies have utilized MLPA, PCR, or WES to identify deletions, which are not sensitive to small breakpoint region differences in the two alleles. Only through long-read sequencing it has become possible to completely characterize these structural variants.

Carriers of single heterozygous pathogenic variants in *SLC12A1*, *SLC12A3*, and *KCNJ1* were shown to have a reduced prevalence of hypertension and for *SLC12A3* also in lower serum potassium levels. A similar effect may also exist in heterozygous Bartter syndrome type 3 carriers [49, 50]. Surprisingly, no such data is available for (unaffected) carriers of causative *CLCNKB* variants and it remains speculative whether the here identified structural variants would exert any (additive) subtle phenotype effects in BS3 carriers. Unaffected family members (BS 3 carriers) were not investigated in this study.

Our deep genotype analysis identified two patients with different *CLCNKA*/*CLCNKB* hybrid genes. Rare *CLCNKA*/*CLCNKB* hybrid genes have been reported previously, but always in a heterozygous state [17, 51, 52]. Here we report two novel hybrid genes in a homozygous state that presumably lead to the expression of hybrid ClC-Ka/ClC-Kb proteins under control of the *CLCNKA* promotor. Complete loss of ClC-Ka and ClC-Kb function would result in hearing loss in addition to salt-wasting tubulopathy (BS 4b) [11]. Interestingly, our patients have no hearing impairment, suggesting an at least partially functional ClC-Ka/ClC-Kb hybrid protein that still can accommodate functions of ClC-Ka. This deduction is further supported by the very high sequence similarity of the individual proteins ClC-Ka and ClC-Kb, with very few AA variations within the transport pathway of the transmembrane region. Although we find district differences between the cytosolic CBS domains in ClC-Ka and ClC-Kb, that is present in the hybrid proteins (Fig. 6A, B), proteins seem to retain partial functionality. Therefore, further work is needed to elucidate the possible functional changes of these differences between ClC-Ka, ClC-Kb, and their hybrid proteins.

In this study, we characterize a novel variant haplotype of the *CLCNKA*/*CLCNKB* genomic region defined by a ~3-kb *CLCNKA* 3′ UTR sequence transposition. This haplotype is significantly associated with structural aberrations in the *CLCNKA*/*CLCNKB* locus and likely represents a predisposing factor for their occurrence. Given an estimated allele frequency of 45–50% in the general population, the frequency of the variant haplotype in our cohort is significantly enriched (*p*=9.16×10^−9^, binominal test). This transposition thus constitutes another example of a common structural polymorphism without an apparent strong influence on gene expression that predisposes to additional genomic rearrangements relevant for human disease as discussed by Poubsky et al. [53].

## Conclusions

In conclusion, we show that the genomic region encompassing *CLCNKA* and *CLCNKB* can give rise to complex structural variants due to high sequence similarity, and emphasize that *CLCNKB* deletions are more diverse than routine diagnostics are able to discriminate. We identify a common haplotype predisposing to *CLCNKB* deletions. Further larger studies are needed to determine whether the precise genomic architecture of the deletion allele has a relevant impact on clinical phenotype and management.

### Supplementary Information

**Additional file 1: Table S1.** Bartter syndrome subtypes and Gitelman syndrome classification. Summary and literature research of the BS subtypes and Gitelman syndrome. **Table S2.** Summary of the molecular genetic analyses performed on each patient. List of molecular genetic analyses performed on each patient. **Table S3.** Summary of the breakpoint region coordinates found in each patient. Breakpoint region coordinates of the eight breakpoint alleles (A-H) including the two sequence transposition haplotypes of 2.2 and 3 kb length, respectively.

**Additional file 2: Fig. S1.** 5 bp deletion confirmed by Sanger Sequencing in patient P18. Sanger Sequencing in patient P18 showing the previously identified 5 bp deletion in exon 9. **Fig. S2.** Schematic view of the Samplix Xdrop custom sequence capture design. Localization of the sequence capture probes in CLCNKA for targeted enrichment of the CLCNKA/CLCNKB locus. **Fig. S3.** T2T CHM13-v.2.0 alignment. Sequence alignment data of the CLCNKA 3‘UTR from three patients, aligned to the T2T CHM13-v2.0 reference genome, illustrating the transposition haplotype structure. **Fig. S4.** Workup of the CLCNKA 3’ UTR sequence transposition haplotype found in this study. CLCNKA 3’ UTR sequence transposition haplotype in short- and long-read whole genome in-house CLCNKB deletion control datasets and public gnomAD database. **Fig. S5.** Gene expression analysis for CLCNKA and CLCNKB. Gene expression analysis for CLCNKA and CLCNKB in renal clear cell carcinoma samples from the Pan-Cancer Atlas database. **Fig. S6.** Long-read Sequencing data. Long-read Sequencing data of the eight different deletion alleles identified in this study visualized in IGV.

## Data Availability

Sequencing alignment data has been deposited in the European Genome-Phenome Archive (https://ega-archive.org/) under the study ID EGAS00001007339 (https://ega-archive.org/studies/EGAS00001007339) [26]. Due to data protection regulations and in accordance with the patient consent, only relevant alignments in the genomic *CLCNKA*/*CLCNKB* locus are shared.

## Abbreviations

BS: Bartter syndrome
BS3: Bartter syndrome type 3
BS 4b: Bartter syndrome type 4b
NAHR: Non-allelic homologous recombination
TAL: Thick ascending limb
DCT: Distal convoluted tubule
GS: Gitelman syndrome
CKD: Chronic kidney disease
MLPA: Multiplex ligation-dependent probe amplification
NGS: Next-generation sequencing
LRS: Long-read third Generation Sequencing
HMW: High molecular weight
EDTA: Ethylenediaminetetraacetic acid
WES: Whole-exome sequencing
WGS: Whole-genome sequencing
CNV: Copy number variation
MAF: Minor allele frequency
ROI: Region of interest
PCR: Polymerase chain reaction
ONT: Oxford Nanopore Technology
ROH: Region of homozygosity
UTR: Untranslated region
SV: Structural variant
AA: Amino-acid
bp: Base-pairs
AF: Alphafold
RMSD: Root-mean-square deviation
