# Supplementary material for: Long-read sequencing identifies a common transposition haplotype predisposing for CLCNKB deletions
Source: Genome Med. 2023 Aug 23;15:62. doi: 10.1186/s13073-023-01215-1 (PMC10464140; doi:10.1186/s13073-023-01215-1)
Supplement: Supplementary file 1 — Additional file 1: Table S1. Bartter syndrome subtypes and Gitelman syndrome classification. Summary and literature research of the BS subtypes and Gitelman syndrome. Table S2. Summary of the molecular genetic analyses performed on each patient. List of molecular genetic analyses performed on each patient. Table S3. Summary of the breakpoint region coordinates found in each patient. Breakpoint region coordinates of the eight breakpoint alleles (A-H) including the two sequence transposition haplotypes of 2.2 and 3 kb length, respectively. [file 13073_2023_1215_MOESM1_ESM.pdf]

| Bartter subtype | Gene | OMIM | Related Protein | Inheritance | prevalence | Clinical phenotype | Biochemical characteristics | Reference |
|---|---|---|---|---|---|---|---|---|
| Type I | *SLC12A1* | 601678 | NKCC2 Na-K_2Cl cotransporter | AR | Rare disease with unknown prevalence. More common BS type. Accounts for 24 % of antenatal BS cases* | Polyhydramnios, preterm delivery, profound postnatal salt wasting, polyuria, risk of acute kidney injury, failure to thrive, nephrocalcinosis | Classic hypokalemic metabolic alkalosis, hypercalciuria, | [6] |
| Type II | *KCNJ1* | 241200 | ROMK Potassium channel | AR | Rare disease with unknown prevalence. More common BS type. Accounts for 25 % of antenatal BS cases* | Polyhydramnios, preterm delivery, postnatal salt wasting, failure to thrive, nephrocalcinosis | As type 1, transient hyperkalemia and metabolic acidosis may occur initially | [7] |

| | | | | | | | | |
|---|---|---|---|---|---|---|---|---|
| Type III | *CLCNKB* | 607364 | ClC-Kb Chloride channel | AR | Rare disease with unknown prevalence. Maybe the most common type of BS worldwide. Accounts for 19 % of antenatal BS cases * | Highly variable onset from antenatal manifestation to school age (GS like BS 3), Polyhydramnios, preterm delivery, polyuria, failure to thrive, nephrocalcinosis | Severe electrolyte disturbances, profound hypokalemia, hypochloremia, variable calcium excretion, hypomagnesemia, | [1, 8] |
| Type IV A | *BSND* | 602522 | Barttin Beta subunit of CLC-Kb and CLC-Ka chloride channels | AR | Rare disease with unknown prevalence. Less common BS type. Accounts for 8 % of antenatal BS cases* | Polyhydramnios, preterm delivery massive postnatal salt wasting, polyuria, failure to thrive, undifferentiated, hyperechogenic kidney | Severe electrolyte disturbances postnatally, variable calcium excretions, Hypomagnesemia, | [9, 10] |

| | | | | | | | | |
|---|---|---|---|---|---|---|---|---|
| | | | | | | parenchyma, early development of chronic renal failure, **hearing loss** | | |
| Type IV B | *CLCNKA* + *CLCNKB* | 613090 | ClC-Ka + ClC-Kb | DR | ultrarare BS type | as type IVa | as type IVa | [11] |
| Type V | *MAGED2* | 300971 | MAGED2 No direct transport | XLR | Rare disease with unknown prevalence. Less common BS type. Accounts for 9 % of antenatal BS cases[a] | Polyhydramnios, preterm delivery, postnatal salt wasting, polyuria, most severe antenatal manifestation, but transient character | Transient hypokalemic metabolic alkalosis, hypercalciuria | [12, 13] |

| | | | | | | | | |
|---|---|---|---|---|---|---|---|---|
| Gitelman | *SLC12A3* | 263800 | NCCT Na-Cl cotransporter | AR | Most common tubulopathy worldwide ca. 1:5000[b] | Variable manifestation, mostly at school age or even incidental diagnosis, fatigue, muscle weakness, tetany, muscle cramps, synkopy | Hypokalemic metabolic alkalosis, hypomagnesemia, hypocalciuria | [4, 14] |

Table S1: Bartter syndrome subtypes and Gitelman syndrome classification: AR, autosomal recessive; DR, digenic recessive; XLR, X-linked recessive;

[a] Prevalence in patients with antenatal BS, reported by Legrand et al. [15]; [b] Estimated incidence of GS reported by Kondo et al. [5].

| **Patient** | **Exome** | **Linked-read** | **Targeted long-fragment enrichment** | **Long-range PCR** | **Long-read WGS (PacBio)** |
|---|---|---|---|---|---|
| P1.1 | Yes | | | Yes (not sequenced) | |
| P1.2 | Yes | Yes | Yes | Yes | |
| P1.3 | Yes | | | Yes | |
| P2 | | | | Yes | |
| P3 | | | | Yes | |
| P4 | | | | Yes | Yes |
| P5 | | | | Yes | |
| P6 | | | | No[a] | Yes |
| P7 | | | Yes | Yes | |
| P8 | | | | Yes | |
| P9 | | | Yes | Yes | |
| P10 | | | | No[a] | Yes |
| P11 | | | Yes | No[a] | |
| P12 | | | | Yes | |
| P13 | | | | Yes | |
| P14 | | | | Yes | |
| P15.1 | | | | Yes | |
| P15.2 | | | | Yes | |
| P16[b] | | | | No[a] | |
| P17 | | | | Yes | |
| P18 | | | | Yes | |

| | | | | | |
|---|---|---|---|---|---|
| P19 | | | | No[a] | |
| P20 | | | | Yes | |
| P21 | | | | Yes | |
| P22 | | | | No[a] | |
| P23 | | | | Yes | |
| P24 | | | | Yes | |
| P25 | | | | No[a] | |
| P26[b] | | | | No[a] | |
| P27 | | | | Yes | |
| P28 | | | | Yes | |
| P29 | | | | Yes | |
| P30 | | | | Yes | |

Table S2: Molecular genetic analyses performed on each patient. [a] Long-range PCR was performed but no PCR fragment could be generated. [b] degraded DNA, low DNA quality.

| **Structural variant** | **Transposition haplotype 2.2kb** | **Transposition haplotype 3.0kb** | **Blue** | **Red** | **Light blue** | **Green** | **Orange** | **Purple** | **Light green** | **Yellow** |
|---|---|---|---|---|---|---|---|---|---|---|
| **Breakpoint coordinates (hg19)** | **chr1:16,360,478_16,362,445del ins[chr1:16,383,736_16,385,874]** | **chr1:16,360,478_16,363,413del ins[chr1:16,383,736_16,386,677]** | **chr1:16,363,803<br>chr1:16,387,061** | **chr1:16,360,478<br>chr1:16,383,736** | **chr1:16,363,772<br>chr1:16,387,030** | **chr1:16,362,445<br>chr1:16,385,874** | **chr1:16,353,336<br>chr1:16,375,142** | **chr1:16,357,595<br>chr1:16,379,321** | **chr1:16,386,677<br>chr1:16,363,413** | **chr1:16,366,847<br>chr1:16,389,355** |
| **P1.1[b]** | X | | X | | | | | | | |
| **P1.2** | X | | X | | | | | | | |
| **P1.3** | X | | X | | | | | | | |
| **P2** | | X | X | | | | | | | |
| **P3** | | X | X | | | | | | | |
| **P4** | | | | | | X | | | | |
| **P5** | | X | | | X | | | | | |
| **P6** | | | | | | | | X | | |
| **P7** | X | | | X | | | | | | |
| **P8** | X | | | X | | | | | | |
| **P9[a] allele 1** | X | | | X | | | | | | |
| **P9[a] allele 2** | | X | | | X | | | | | |
| **P10** | X | | | | | | | | | X |
| **P11** | | | X | | | | X | | X | |
| **P12** | | X | X | | | | | | | |
| **P13** | | X | X | | | | | | | |
| **P14[a] allele 1** | X | | | X | | | | | | |
| **P14[a] allele 2** | | X | | | X | | | | | |
| **P15.1** | | | | | | X | | | | |
| **P15.2** | | | | | | X | | | | |
| **P17** | X | | | X | | | | | | |
| **P18[a]** | | X | | | X | | | | | |
| **P20** | X | | | X | | | | | | |

| | | | | | | | | | | |
|---|---|---|---|---|---|---|---|---|---|---|
| **P21** | | X | X | | | | | | | |
| **P23** | | X | | | X | | | | | |
| **P24** | | X | X | | | | | | | |
| **P27** | | X | X | | | | | | | |
| **P28** | | X | X | | | | | | | |
| **P29** | | X | X | | | | | | | |
| **P30** | X | | | X | | | | | | |

Table S3: Breakpoint-region coordinates found in this study. For easier readability, structural variants with ambiguous breakpoints inside homologous regions are described in accordance with the HGVS Recommendations for Sequence Variant Nomenclature, whereby the breakpoint is arbitrarily assigned to the 3'-most position allowed by the sequence context (3' rule). Colors listed behind the breakpoint coordinates refer to the colors used in Figure 4. [a] structural variant in compound heterozygosity. [b] No long-read sequencing done, but short-read WES shows identical genotype as both affected brothers.
