# Supplementary material for: Long-read sequencing identifies a common transposition haplotype predisposing for CLCNKB deletions
Source: Genome Med. 2023 Aug 23;15:62. doi: 10.1186/s13073-023-01215-1 (PMC10464140; doi:10.1186/s13073-023-01215-1)
Supplement: Supplementary file 2 — Additional file 2: Fig. S1. 5 bp deletion confirmed by Sanger Sequencing in patient P18. Sanger Sequencing in patient P18 showing the previously identified 5 bp deletion in exon 9. Fig. S2. Schematic view of the Samplix Xdrop custom sequence capture design. Localization of the sequence capture probes in CLCNKA for targeted enrichment of the CLCNKA/CLCNKB locus. Fig. S3. T2T CHM13-v.2.0 alignment. Sequence alignment data of the CLCNKA 3‘UTR from three patients, aligned to the T2T CHM13-v2.0 reference genome, illustrating the transposition haplotype structure. Fig. S4. Workup of the CLCNKA 3’ UTR sequence transposition haplotype found in this study. CLCNKA 3’ UTR sequence transposition haplotype in short- and long-read whole genome in-house CLCNKB deletion control datasets and public gnomAD database. Fig. S5. Gene expression analysis for CLCNKA and CLCNKB. Gene expression analysis for CLCNKA and CLCNKB in renal clear cell carcinoma samples from the Pan-Cancer Atlas database. Fig. S6. Long-read Sequencing data. Long-read Sequencing data of the eight different deletion alleles identified in this study visualized in IGV. [file 13073_2023_1215_MOESM2_ESM.pdf]

Control

Patient P18

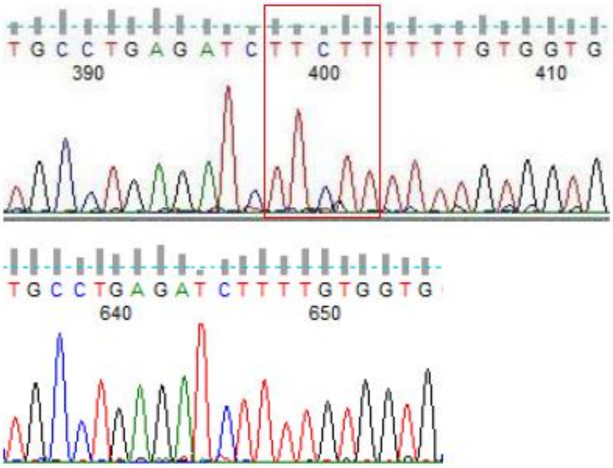

Fig. S1. 5 bp deletion confirmed by Sanger Sequencing in patient P18.

Sanger Sequencing of *CLCNKB* exon 9 in P18 (lower panel) and a control (upper panel). The 5 bp deletion appears homozygous because of the *CLCNKB* deletion allele F *in trans*.

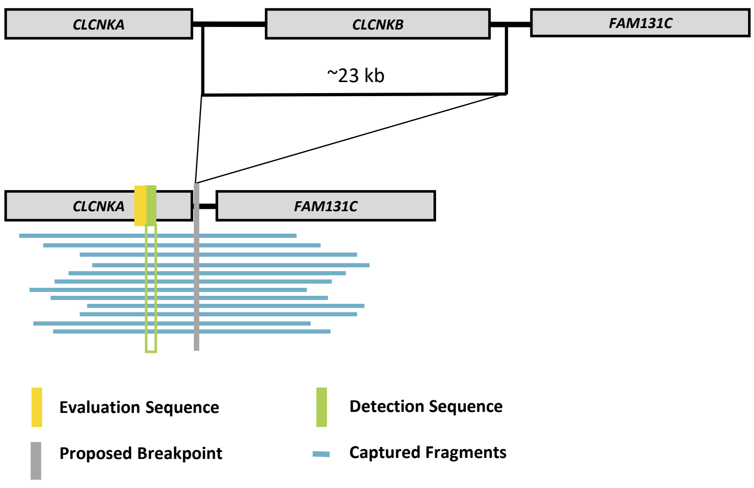

Fig. S2. Schematic view of the Samplix Xdrop custom sequence capture design.

Detection and evaluation sequence designed in *CLCNKA* to capture up to 50 kb of HMW genomic DNA fragments covering *CLCNKA* and *CLCNKB*. Proposed breakpoints in the region between *CLCNKA* and *CLCNKB*.

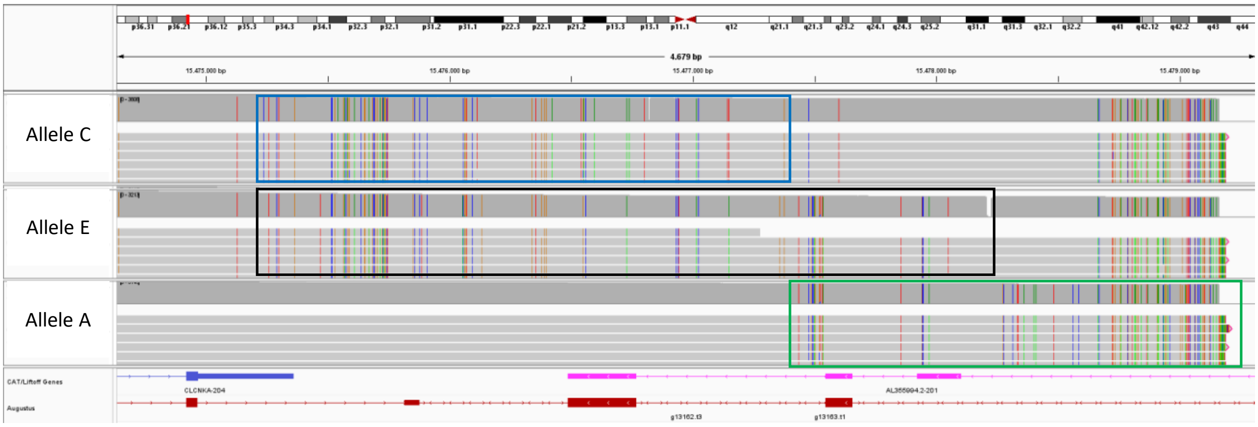

Fig. S3. T2T CHM13-v.2.0 alignment

Sequence alignment data of the *CLCNKA* 3‘UTR from three patients, aligned to the T2T CHM13-v2.0 reference genome, illustrating the transposition haplotype structure. The smaller ~2.2 kb sequence transposition haplotype is indicated by the blue box. The larger ~3 kb sequence transposition is indicated by the black box. The *CLCNKB* sequence after the single breakpoint region in allele A (allele without transposition haplotype) is indicated by the green box.

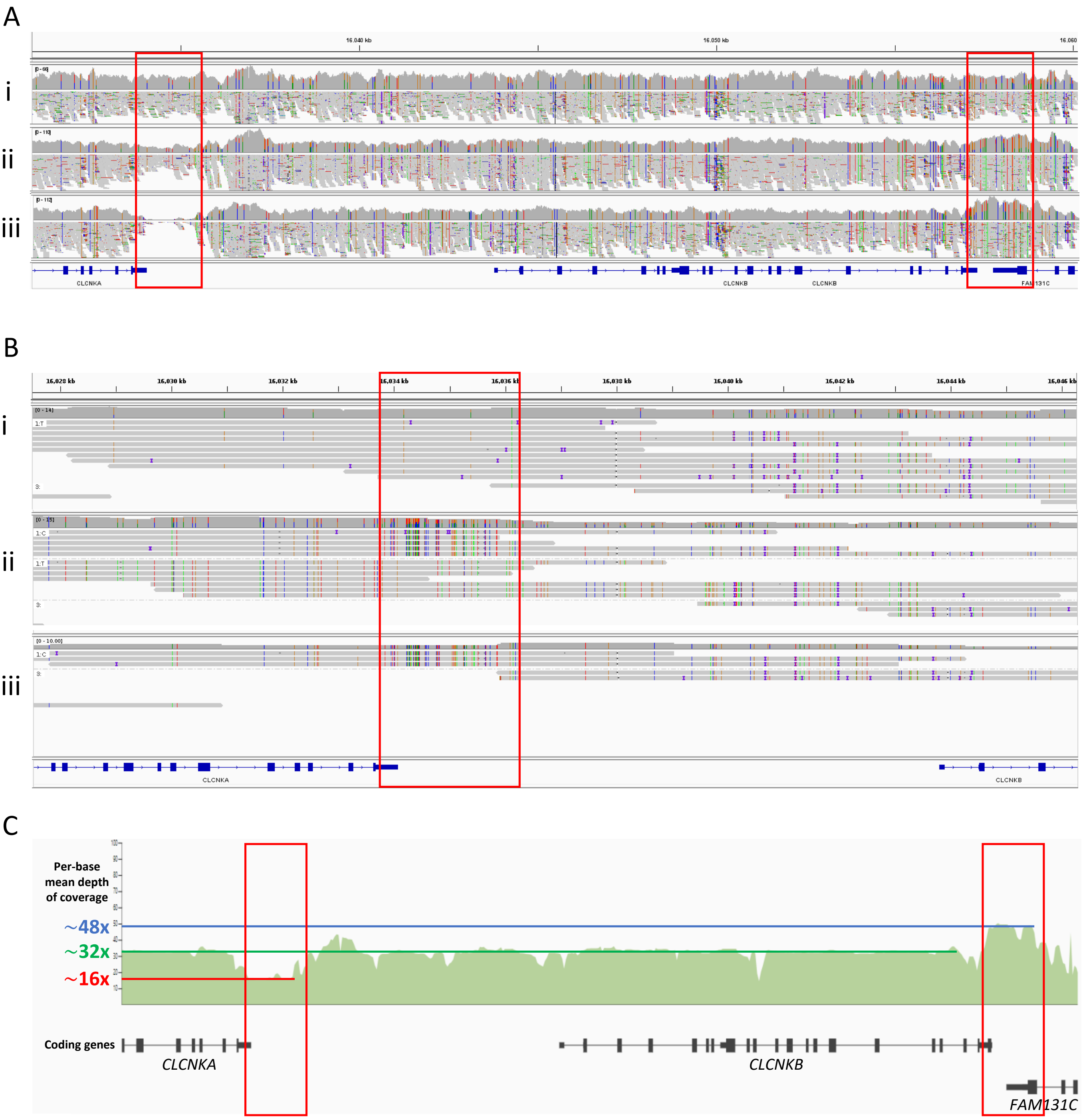

A
i
ii
iii
16.040 kb
16.050 kb
16.060
CLCNKA
CLCNKB
CLCNKB
FAM131C
B
16,028 kb
16,030 kb
16,032 kb
16,034 kb
16,036 kb
16,038 kb
16,040 kb
16,042 kb
16,044 kb
16,046 kb
CLCNKA
CLCNKB
C
Per-base mean depth of coverage
~48x
~32x
~16x
Coding genes
CLCNKA
CLCNKB
FAM131C

Fig. S4. Workup of the *CLCNKA* 3' UTR sequence transposition haplotype found in this study.

CLCNKA 3' UTR sequence transposition haplotype in short- and long-read whole genome in-house *CLCNKB* deletion control datasets. Whole genome data of the reference (i) and the heterozygous (ii) and homozygous (iii) variant haplotype, respectively. Sequences are visualized in IGV. Genes are indicated in blue; Exons are indicated as vertical bars; Gene orientation is indicated by arrows. Genomic coordinates refer to the hg38 human reference. **A** In the short-read alignment, the duplicated 2.2-3 kb 3' UTR of *CLCNKB* are aligned in the *CLCNKB* 3' UTR (right red square), leading to a coverage gain in the variant haplotype carriers. The loss of the 3' UTR of *CLCNKA* is indicated by the reduced coverage (left red square). **B** In the long-read alignment, the inserted *CLCNKB* 3' UTR fragment (red square) is indicated by the differentiating nucleotides that distinguish the *CLCNKA* 3' UTR and the *CLCNKB* 3' UTR. **C** Coverage plot of the *CLCNKA*/*CLCNKB* gene locus adapted from gnomAD (3.1.2). Reduced coverage depth in the *CLCNKA* 3' UTR (left red square) and elevated coverage in the *CLCNKB* 3' UTR (right red square) are visible in the normal population data. This data indicates a 2.2-3 kb loss of *CLCNKA* 3' UTR sequence and a duplication of the *CLCNKB* 3' UTR sequence in approximately 50 % of control alleles, corresponding to the expected representation of the transposition haplotype in short-read NGS data.

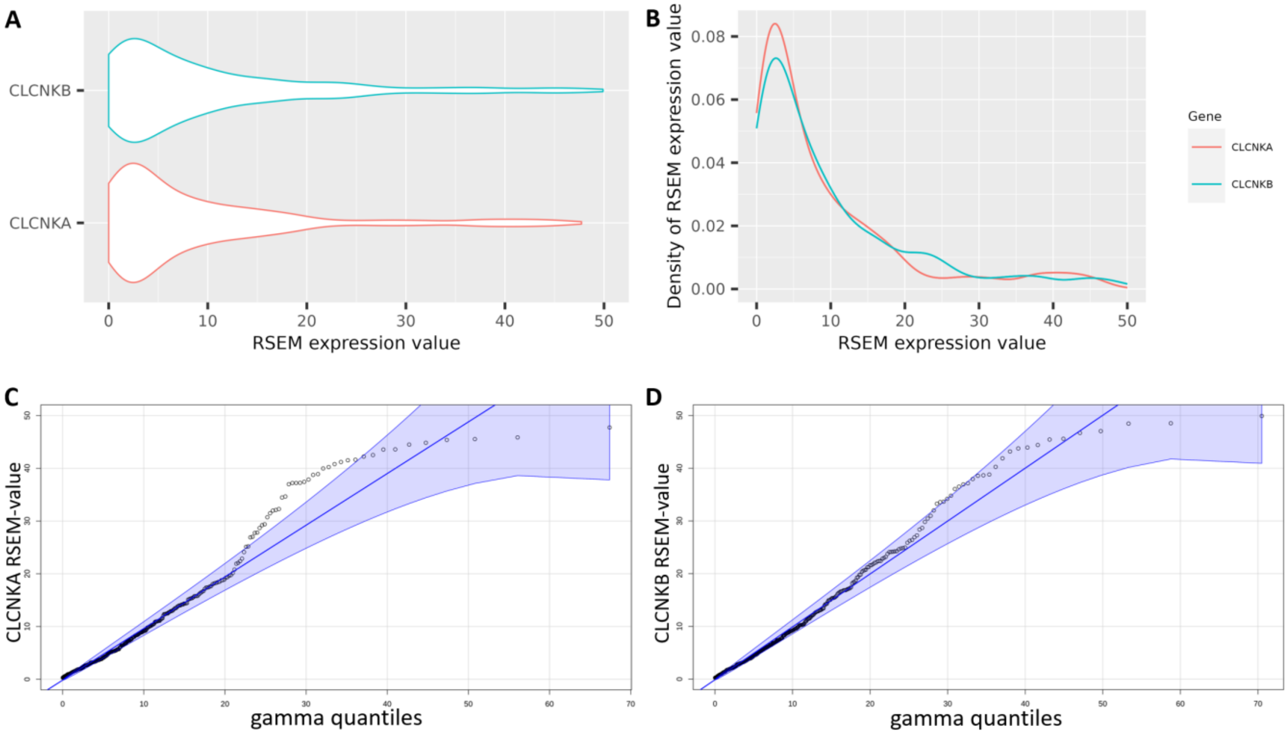

Fig. S5. Gene expression analysis for *CLCNKA* and *CLCNKB*

Gene expression analysis for *CLCNKA* and *CLCNKB* in renal clear cell carcinoma samples from the Pan-Cancer Atlas database (n=510). **A** Violin plot of expression levels in *CLCNKA* and *CLCNKB* between RSEM values of 0 and 50 (83.1% and 81.2% of observations for *CLCNKA* and *CLCNKB*, respectively). **B** RSEM expression value density plot for *CLCNKA* (red) and *CLCNKB* (blue) between RSEM values of 0 and 50. **C** Q-Q plot of *CLCNKA* RSEM expression values between 0 and 50 (zero values excluded) for the closest fit gamma distribution (p=0.0504). **D** Q-Q plot of *CLCNKB* RSEM expression between 0 and 50 (zero values excluded) for the closest fit gamma distribution (p=0.4076). The p-values for goodness-of-fit for a normal and lognormal distribution (Q-Q plots not shown) were all less than $1x10^{-5}$, hence the gene expression was deemed unlikely to correspond to these distribution categories.

Deletion allele A

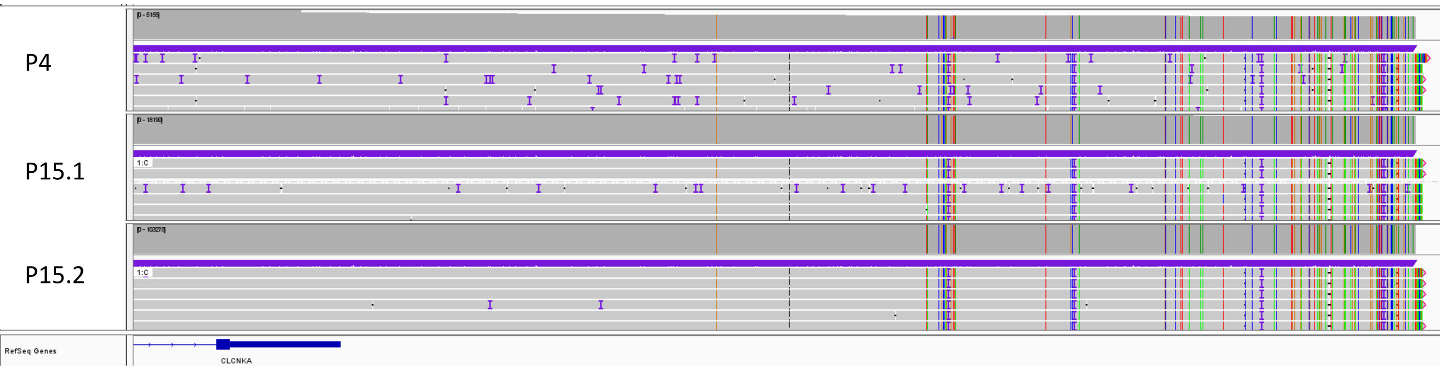

Deletion allele B

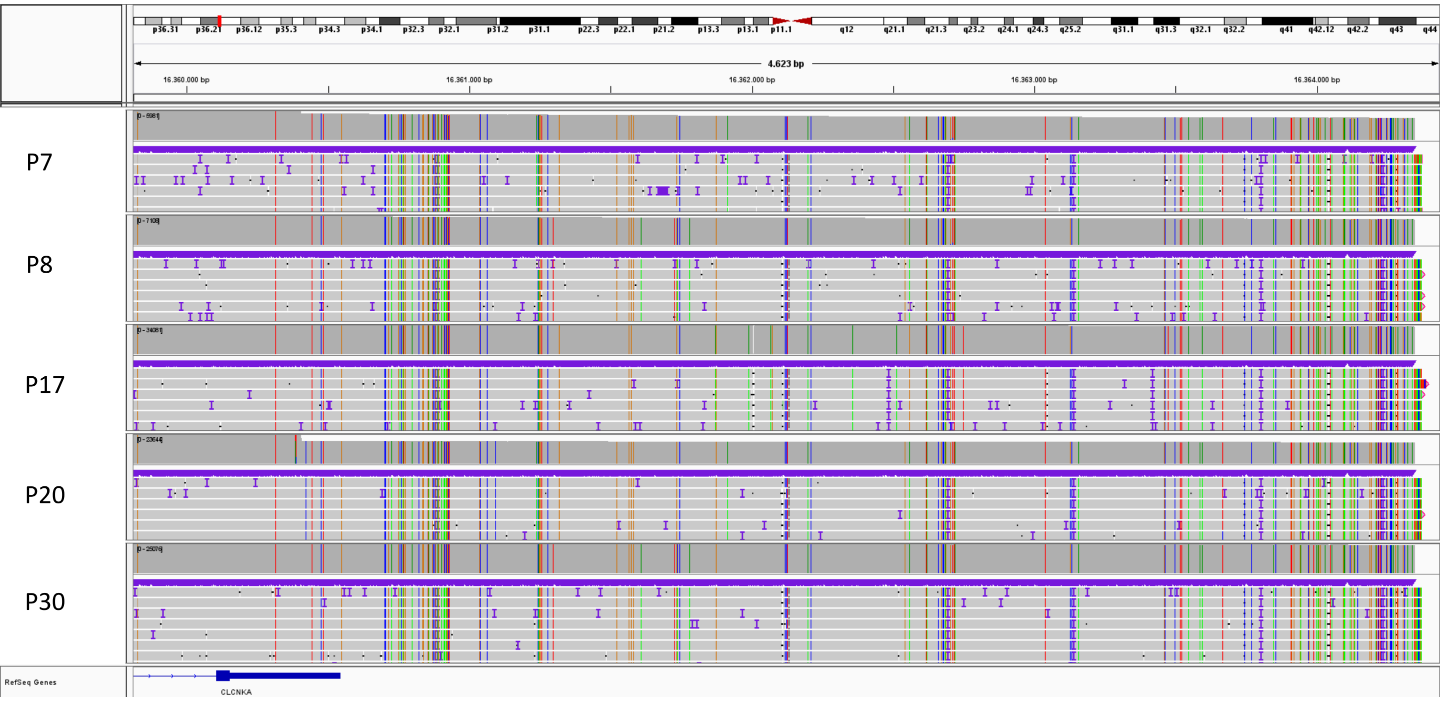

Deletion allele C

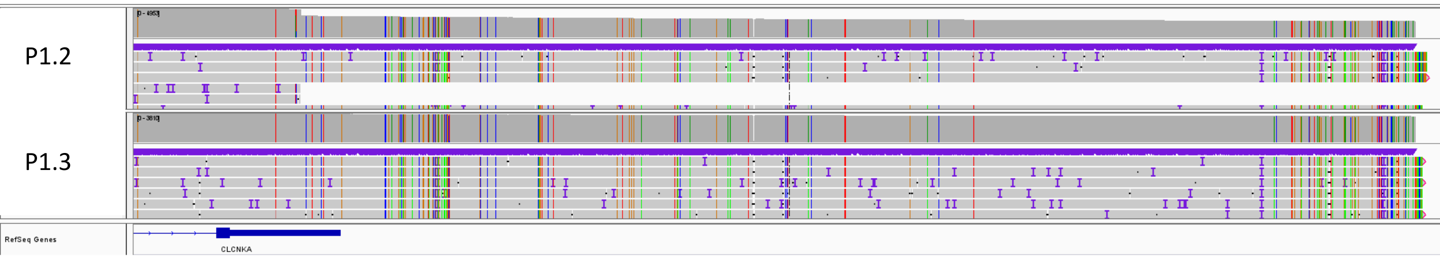

Deletion allele D

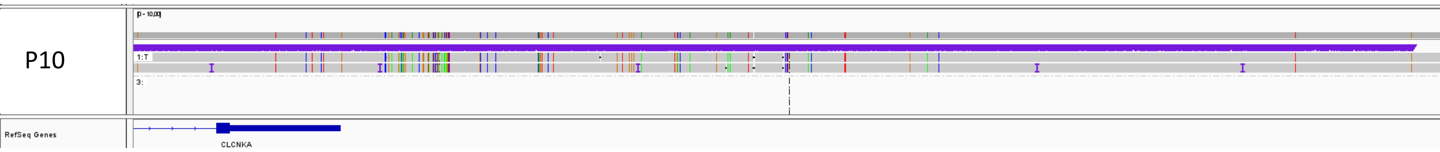

Deletion allele E

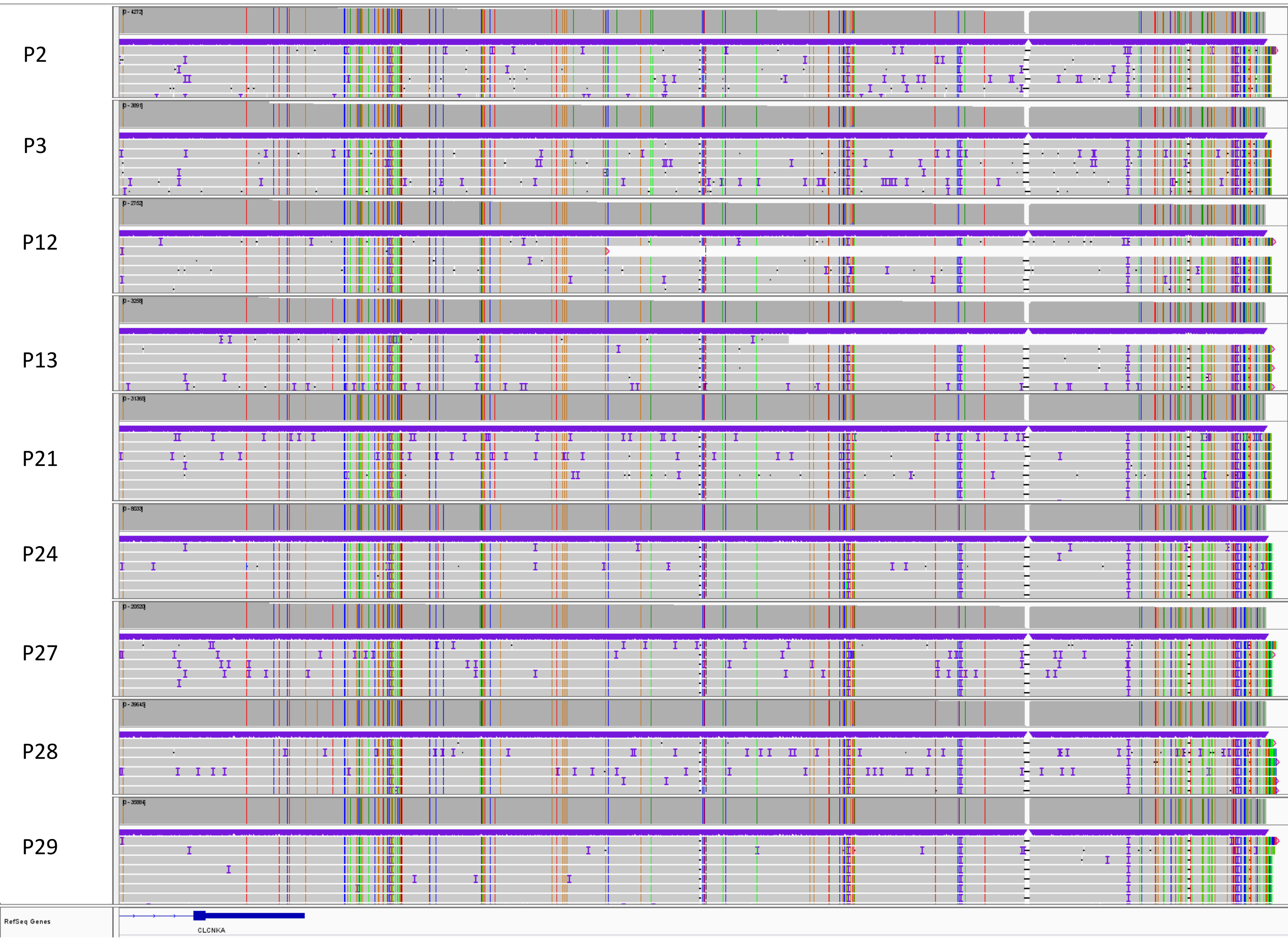

Deletion allele F

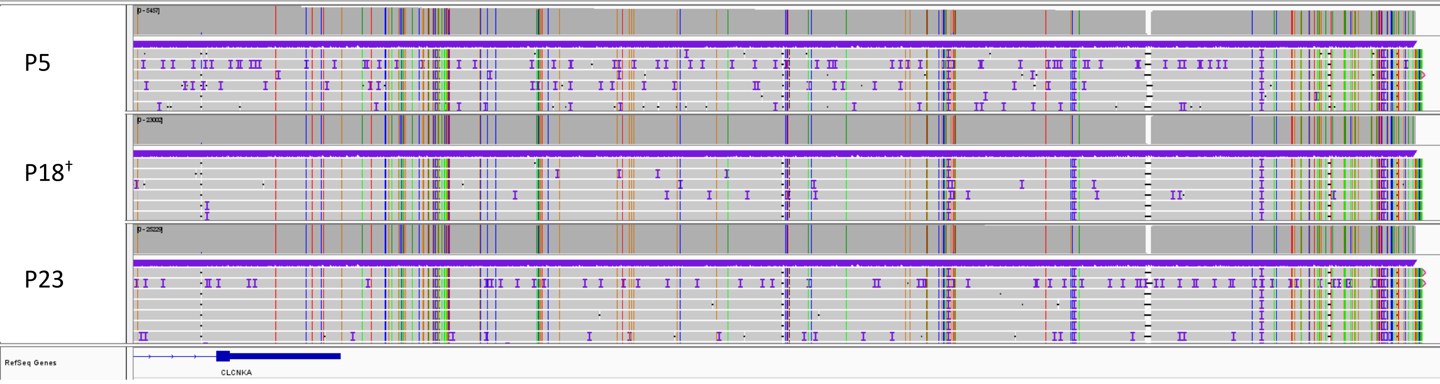

Deletion allele B + F

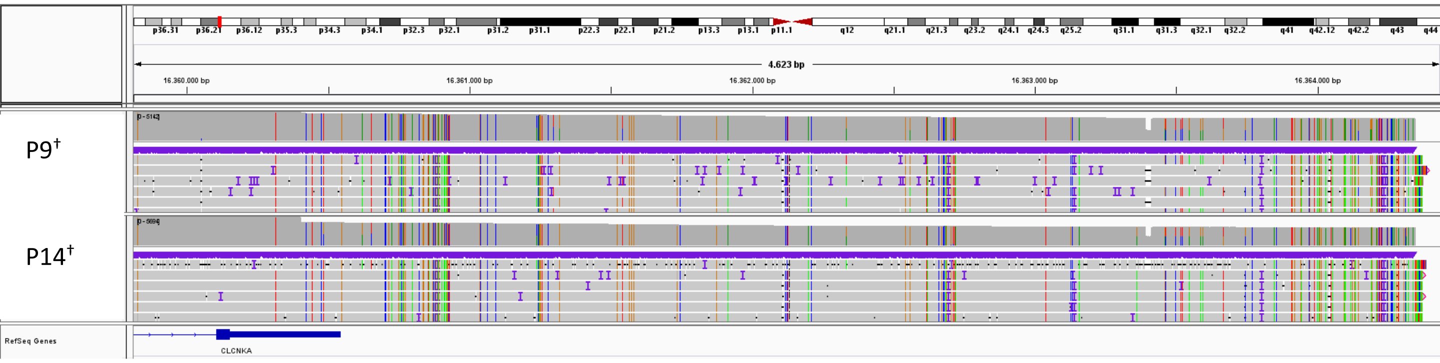

Deletion allele G

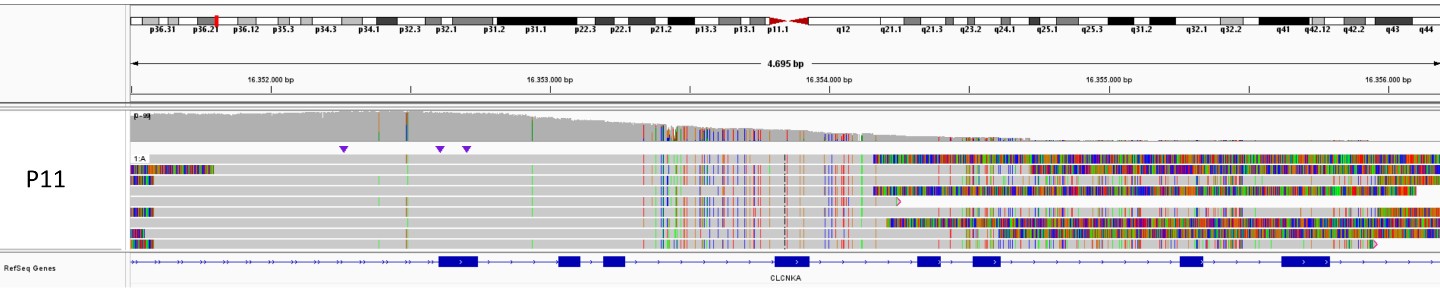

Deletion allele H

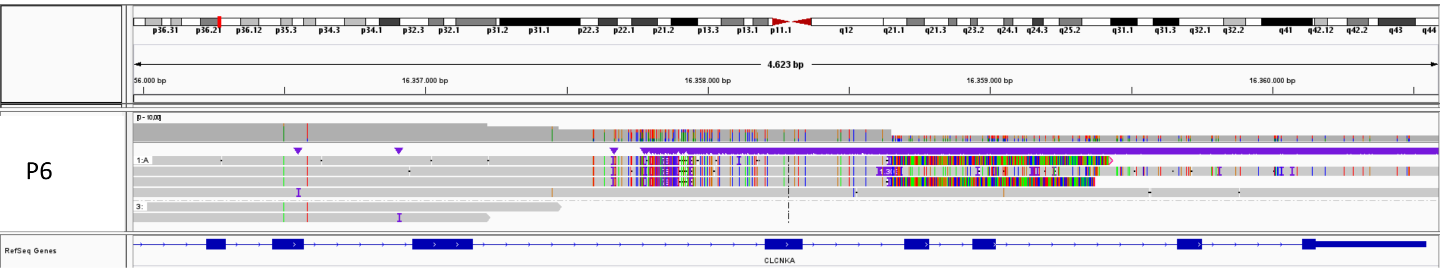

Fig. S6. Long-read Sequencing data.

Long-read Sequencing data of eight different deletion alleles (A-H) identified in 28 patients from this study visualized in IGV [26].
